# *SciPhon*: a data analysis software for nuclear resonant inelastic X-ray scattering with applications to Fe, Kr, Sn, Eu and Dy

**DOI:** 10.1107/S1600577518009487

**Published:** 2018-08-21

**Authors:** Nicolas Dauphas, Michael Y. Hu, Erik M. Baker, Justin Hu, Francois L. H. Tissot, E. Ercan Alp, Mathieu Roskosz, Jiyong Zhao, Wenli Bi, Jin Liu, Jung-Fu Lin, Nicole X. Nie, Andrew Heard

**Affiliations:** a Department of the Geophysical Sciences and Enrico Fermi Institute, The University of Chicago, 5734 South Ellis Avenue, Chicago, IL 60615, USA; b Advanced Photon Source, Argonne National Laboratory, 9700 South Cass Avenue, Argonne, IL 60439, USA; c Department of Earth and Planetary Sciences, Northwestern University, 2145 Sheridan Road, Evanston, IL 60208, USA; d IMPMC-UMR CNRS 7590, Sorbonne Universités, UPMC, IRD, MNHN, Muséum National d’Histoire Naturelle, 61 Rue Buffon, 75005 Paris, France; e Department of Geological Sciences, Stanford University, Stanford, CA, USA; f Department of Geological Sciences, Jackson School of Geosciences, University of Texas at Austin, Austin, TX 78712, USA

**Keywords:** NRIXS, GUI software, data reduction, Mossbauer, lattice dynamics

## Abstract

The synchrotron radiation technique of nuclear resonant inelastic X-ray scattering (NRIXS), also known as nuclear resonance vibrational spectroscopy or nuclear inelastic scattering, provides a wealth of information on the vibrational properties of solids. It has found applications in studies of lattice dynamics and elasticity, superconductivity, heme biochemistry, seismology, isotope geochemistry and many other fields. It involves probing the vibrational modes of solids by using the nuclear resonance of Mössbauer isotopes such as ^57^Fe, ^83^Kr, ^119^Sn, ^151^Eu and ^161^Dy. After data reduction, it provides the partial phonon density of states of the Mössbauer isotope that is investigated, as well as many other derived quantities such as the mean force constant of the chemical bonds and the Debye velocity. The data reduction is, however, not straightforward and involves removal of the elastic peak, normalization and Fourier–Log transformation. Furthermore, some of the quantities derived are highly sensitive to details in the baseline correction. A software package and several novel procedures to streamline and hopefully improve the reduction of the NRIXS data generated at sector 3ID of the Advanced Photon Source have been developed. The graphical user interface software is named *SciPhon* and runs as a *Mathematica* package. It is easily portable to other platforms and can be easily adapted for reducing data generated at other beamlines. Several tests and comparisons are presented that demonstrate the usefulness of this software, whose results have already been used in several publications. Here, the *SciPhon* software is used to reduce Kr, Sn, Eu and Dy NRIXS data, and potential implications for interpreting natural isotopic variations in those systems are discussed.

## Abstract 2

*SciPhon* is a software for the reduction of nuclear resonant inelastic X-ray scattering data. Tests and examples of applications to Fe, Kr, Sn, Eu and Dy data are presented.

## 1. Introduction   

The method of nuclear resonant inelastic X-ray scattering [NRIXS; also known as nuclear resonance vibrational spectroscopy (NRVS) or nuclear inelastic scattering (NIS)] is a synchrotron radiation technique that allows one to probe the vibrational properties of a solid (Singwi & Sjölander, 1960 ▸; Visscher, 1960 ▸; Sturhahn *et al.*, 1995 ▸; Alp *et al.*, 2002 ▸; Sturhahn, 2004 ▸; Chumakov & Sturhahn, 1999 ▸; Kohn *et al.*, 1998 ▸). Despite its relative recent development, it has already found important applications in a variety of scientific fields. In geophysics, it is used to derive acoustic wave velocities, which are critical to interpret seismograms from which the internal structure and composition of the Earth can be inferred (Mao *et al.*, 2001 ▸; Hu *et al.*, 2003 ▸; Mao *et al.*, 2006 ▸; Lin *et al.*, 2003 ▸, 2013 ▸, 2014 ▸; Sturhahn & Jackson, 2007 ▸). In geochemistry, the mean force constants of chemical bonds can be measured, allowing one to predict how isotopes should partition between coexisting phases at equilibrium (Polyakov *et al.*, 2005 ▸, 2007 ▸; Polyakov, 2009 ▸; Dauphas *et al.*, 2012 ▸, 2014 ▸; Blanchard *et al.*, 2015 ▸; Roskosz *et al.*, 2015 ▸; Shahar *et al.*, 2016 ▸). In condensed matter physics and material sciences, the partial phonon density of states of Mössbauer isotopes provide considerable insights into lattice dynamics and related properties of the materials (Röhlsberger, 2004 ▸). In biochemistry, the vibration modes provide clues on the arrangement of ligands around the heme group (Sage *et al.*, 2001 ▸; Scheidt *et al.*, 2005 ▸, 2017 ▸). A common feature of NRIXS usage across all these fields is that the measurements always take time and are technically challenging. Developing refined and rapid data reduction tools is thus critical to make the most efficient use of this technique and the limited beam time available for NRIXS measurements at synchrotrons.

The method of NRIXS uses the excitation of Mössbauer isotopes to probe the vibration properties of solids (Sturhahn *et al.*, 1995 ▸; Seto *et al.*, 1995 ▸; Alp *et al.*, 2002 ▸; Sturhahn, 2004 ▸; Chumakov & Sturhahn, 1999 ▸; Kohn *et al.*, 1998 ▸). In the following, we will take ^57^Fe as an example and describe the experimental setup used at sector 3ID of the Advanced Photon Source (APS) at Argonne National Laboratory. Other Mössbauer isotopes such as ^83^Kr, ^119^Sn, ^151^Eu and ^161^Dy are routinely measured by NRIXS at the APS and other beamlines around the world. The software introduced here is applicable to those systems as well. The Mössbauer isotope ^57^Fe has a low-lying nuclear excited state at 14.4125 keV. The approach used in NRIXS is to scan the energy around this transition and measure X-rays scattered by the de-excitation of the ^57^Fe nuclei. The incident X-rays are monochromated to 1 meV band-pass FWHM (full width at half-maximum). They consist of pulses of 70 ps duration separated by interpulses of 153 ns duration. All electronic X-ray scattering processes including elastic Thomson scattering, Compton scattering and possible fluorescence emissions are instantaneous and rapidly decay. The excited state of ^57^Fe has a lifetime of 141 ns so the X-rays scattered by nuclear excitation are emitted with a delay. By applying appropriate time discrimination, and only collecting the signal emitted during the inter-pulse period, it is possible to eliminate X-rays from electronic scattering and only consider those produced by nuclear resonant scattering. The energy of the incident X-ray beam is scanned around the nominal nuclear resonance energy of ^57^Fe (*i.e.* 14.4125 keV) and the scattered X-rays are collected (in the case of ^57^Fe, it is advantageous to record the 6.403 keV *K*_α1_, 6.391 keV *K*_α2_ and 7.057 keV *K*_β_ iron fluorescence signal induced by internal conversion because the yield is higher and the efficiency of the detector is increased). The plot of delayed nuclear resonance signal *versus* energy (*E*) after proper normalization is called the phonon excitation probability density and is denoted 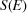. When the incident X-rays have lower energy than the nominal nuclear resonance energy, excitation of ^57^Fe can also occur if lattice vibrations (or their particle-like equivalents phonons) provide the missing energy in a process known as phonon annihilation. Conversely, if the incident X-rays have higher energy than the nominal resonance energy, excitation of ^57^Fe can still occur if lattice vibrations can absorb the extra energy in a process known as phonon creation. The phonon excitation probability density function 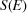 thus contains considerable information on lattice vibrations, the macroscopic manifestations of which are the elastic properties of the material considered. In particular, appropriate data processing yields the phonon density of states (PDOS) (Sturhahn *et al.*, 1995 ▸). The PDOS is partial in that iron (or any other resonant nuclide; Diakhate *et al.*, 2011 ▸; Simon *et al.*, 2014 ▸; Long *et al.*, 2005 ▸) is the only nucleus that is probed by the technique, and it is projected in that the quantities derived are projected along the direction of the incident beam (Chumakov *et al.*, 1997 ▸; Kohn *et al.*, 1998 ▸). For isotropic materials such as cubic crystals, the PDOS has no directionality. For powder, the PDOS is averaged over all directions.

The first NRIXS measurements were reported in 1995 (Seto *et al.*, 1995 ▸) and it was immediately recognized that the iron PDOS could be derived from such measurements (Sturhahn *et al.*, 1995 ▸). The natural abundance of ^57^Fe is only 2.119% of total iron so the measurements are most often made on synthetic materials that have been enriched in ^57^Fe. This cuts on acquisition time but NRIXS remains a time-intensive technique, as it uses a very high resolution monochromator that also reduces the flux of X-rays. A good spectrum can be acquired in a time span of hours to days. Several synchrotron beamlines around the world can perform NRIXS measurements (sectors 3ID, 16-ID and 30-ID at APS, USA; ID-18 at ESRF, France; P01 at PETRA-III, Germany; and BL09XU and BL11XU at SPring-8, Japan). The analysis of NRIXS data is quite involved. To make most efficient use of precious synchrotron beam time, it is important to develop data processing software such as *PHOENIX* (Sturhahn, 2000 ▸) or *DOS* (Kohn & Chumakov, 2000 ▸) that allow beamline users to analyze the results concurrently with data acquisition so that they can assess if sufficient counts have been acquired or if the measurements suffer from biases that can be rapidly addressed. The software of choice for reducing NRIXS data at sector 3ID of the APS is *PHOENIX* (Sturhahn, 2000 ▸), which is written in Fortran90 and is a command-line based program. To streamline data reduction (Blanchard *et al.*, 2015 ▸; Dauphas *et al.*, 2014 ▸), we have developed a new software for NRIXS analysis to meet the following requirements:

(i) It has a graphical user interface (GUI), which facilitates learning of the program and speeds up data processing for the most repetitive tasks.

(ii) It provides flexibility in the definition of the baseline.

(iii) It allows the user to define the energy range used in data reduction.

(iv) It propagates uncertainties not only from counting statistics but also from baseline subtraction and energy scaling.

(v) It outputs all the parameters already given by *PHOENIX*.

The program is named *SciPhon* (*Science of Phonons*) and is a package that can be run under *Mathematica*. The reason why *Mathematica* was used is that it makes it easy to develop a GUI that is portable from one operating system to another and from one software generation to another. The software is available from the corresponding author upon request, from scientists at sector 3ID of the APS, and from the website http://originslab.uchicago.edu/Software-and-Facilities.

## 2. *SciPhon* operation   

**Figure 1:**
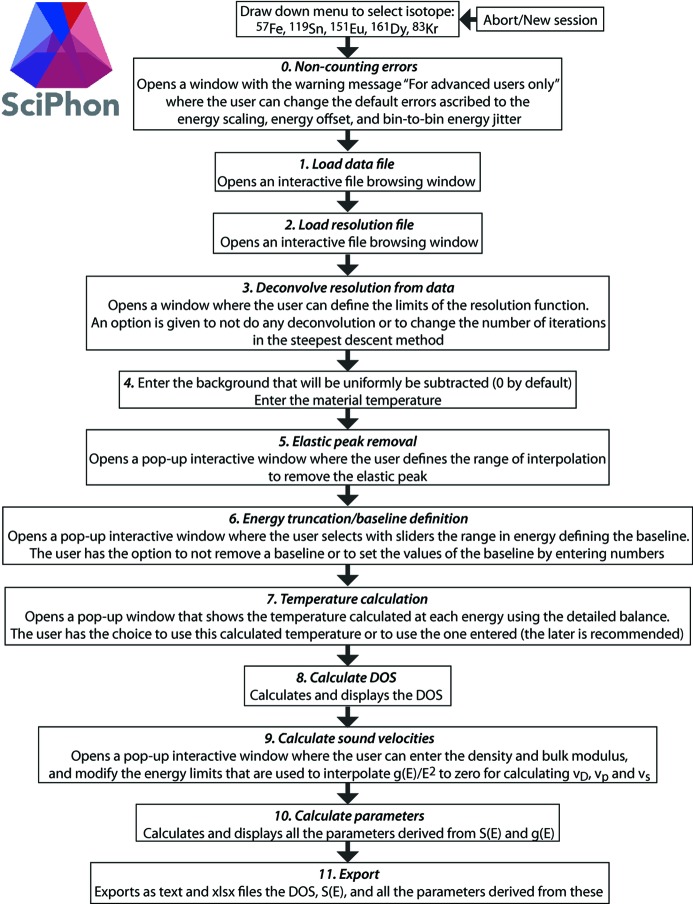
Flowchart of the *SciPhon* *Mathematica* package. Fig. 3 ▸ shows how these actions are implemented through GUI display panels. See text for details.

**Figure 2:**
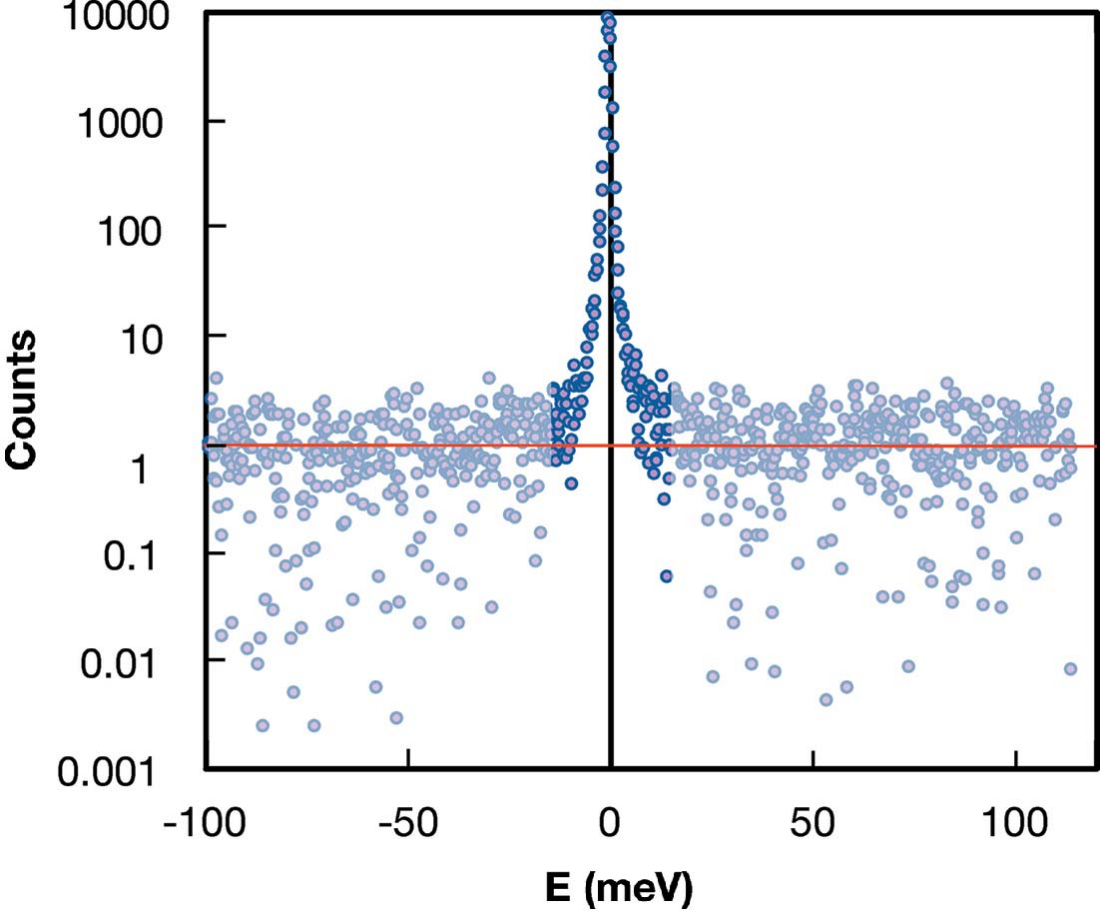
Example of the resolution function measured in the nuclear forward-scattering channel. The FWMH is ∼1 meV. The intensity decreases by four orders of magnitude in the first ±15 meV. Beyond that, the measured counts are below baseline level. Before further processing, the baseline counts (in red here) are subtracted form the signal and the spectrum is truncated to eliminate the low- and high-energy tails where the signal is below baseline (here beyond ∼±15 meV).

Below, we provide a step-by-step explanation of the algorithm behind *SciPhon* (Fig. 1 ▸). The inputs are the measured phonon spectrum and the resolution function. The NRIXS signal is measured by one or several avalanche photodiode (APD) detectors that are positioned, on the incident beam side, as close as possible to the sample. The forward-scattering signal corresponds to a convolution of the natural linewidth of the 14.4125 keV transition of ^57^Fe (4.66 neV) and the resolution function of the monochromator (∼1.0 meV FWHM). Because the latter is so much larger than the former, the signal formed by nuclear forward-scattered X-rays is a proxy for the resolution function of the monochromator (Fig. 2 ▸). This signal is measured by one APD detector placed far away behind the sample in the direction of the beam. For samples that are too thick for X-rays to pass through, a resolution function measured during the same session as the samples should be used. The data and resolution files can be obtained using the ‘padd’ module, which is part of *PHOENIX* (Sturhahn, 2000 ▸). Padd stacks the different scans that belong to a single sample and establishes the energy scale. A typical NRIXS scan would be from −130 to +150 meV in steps of 0.25 meV. To minimize bias introduced by unaccounted drift (increase or decrease) in the instrument response with time, we alternate between low to high (−130 to +150 meV) and high to low (+150 to −130 meV) energy scans. The expectation is that by measuring an even number of scans the systematic effects associated with such a change in the instrument response can be minimized.

### 2.1. Selection of a Mössbauer isotope   

**Figure 3:**
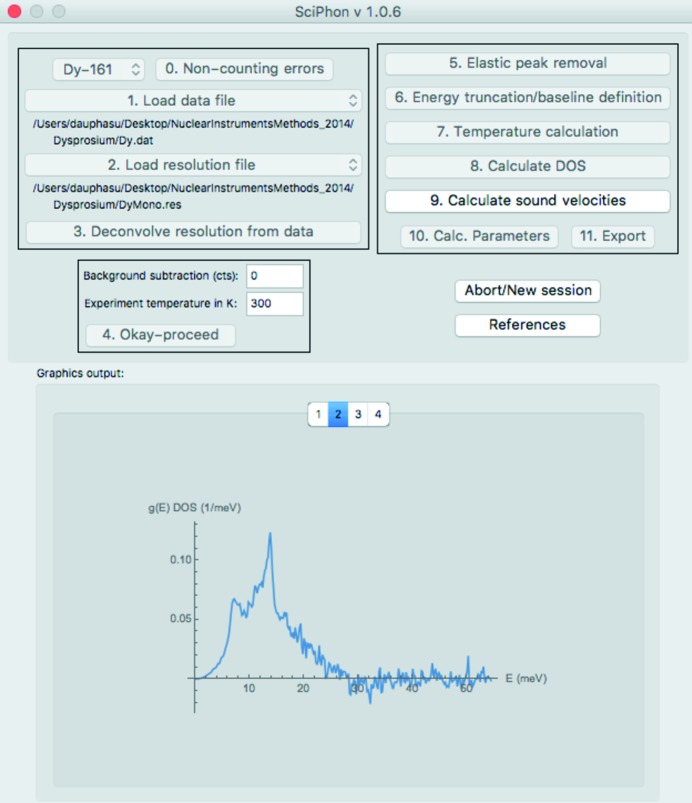
Main GUI display panel of *SciPhon*. Fig. 1 ▸ shows the sequence of actions that call the opening of these windows. See text for details.

A picture of the GUI is shown in Fig. 3 ▸. The user starts by selecting the Mössbauer isotope that was measured by NRIXS. The options given in a drop-down menu are ^57^Fe, ^119^Sn, ^151^Eu, ^161^Dy and ^83^Kr, with ^57^Fe selected as the default option. The user is then guided through a sequence of buttons numbered 1 to 11 that all perform a task. The buttons appear as gray and cannot be clicked before all the tasks needed for that action are completed. Once a button is clicked and an action is performed, the button turns gray and cannot be clicked again. If a mistake is made, the user has the option of aborting the sequence and starting the data reduction anew.

### 2.2. Load data file   

The data file contains a header where each line starts with the symbol # and which is discarded by *SciPhon*. The rest of the file contains three columns. The first column is the energy in meV. The second column is the total number of counts in each energy channel. The monochromator does not produce a perfectly flat intensity profile as a function of energy and the intensity provided by the synchrotron can fluctuate. These variations are corrected for in padd (part of the *PHOENIX* software) by normalizing the counts to the flux measured in an ionizing chamber (IC1) located after the monochromator. The third column in the data file is the 1σ error from counting statistics (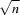, where *n* is the number of counts in each channel). The user selects the data file by using a standard file browser interface. The directory where the data file is located is the default output directory for *SciPhon*, where the results of the calculation are exported.

### 2.3. Load resolution file   

The resolution function file contains a header that is discarded. The first column records the energy in meV, the second column contains the counts, and the third column contains the uncertainty from counting statistics. The uncertainty column is not used in the calculation. By default, the software opens the file browser window starting in the directory where the data file is, but the user can then browse other directories to find the resolution function.

### 2.4. Deconvolution of the resolution function and spectrum   

In samples that do not block X-rays, the resolution function 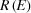 is measured in the forward channel at the same time as the NRIXS signal is being measured. It thus has the same energy range as the samples. In samples that are too thick to let X-rays pass through, it can be separately measured in the forward channel before or after the sample measurement. In samples that do not block X-rays, 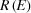 is measured over the same energy range as the data. The resolution function typically decreases to values that are below background outside of about −15 to +15 meV (Fig. 2 ▸). In *SciPhon*, the user is thus given the option to truncate the resolution function and only use the potion that is above background level. This is done using an interactive graphics interface where 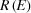 is plotted as a function of *E* on a log–linear scale. The user can use this plot to visually assess the energy values beyond which the signal is all background and to set those limits using vertical sliders. Once those energies have been set, *SciPhon* truncates 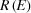 to this range and a baseline is subtracted by interpolating the background signal measured outside the truncation range. The resolution function thus defined (truncated and with a baseline subtracted) is denoted 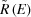 hereafter.

The next step in the algorithm is the optional deconvolution of 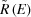 from 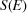. One may wonder why this deconvolution is done before baseline subtraction on 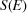. Convolution has the property of distributivity, meaning that deconvolution of the signal and the baseline can be examined independently. The convolution of an even function whose integral is 1 (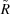 is normalized and is approximately symmetric around zero energy) with a linear function is the linear function itself. This means that it would make no difference if the baseline was subtracted before or after peak deconvolution. This being true, we prefer to carry out the peak deconvolution first because some peak deconvolution algorithms do not handle well negative numbers that can arise when a background/baseline is subtracted.

The measured NRIXS spectrum is a convolution of the ‘true’ spectrum and the resolution function, 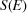 = 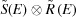. A mathematical deconvolution can be performed by dividing the discrete Fourier transform of 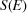 by the Fourier transform of 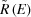, and then taking the inverse Fourier transform of that ratio. While mathematically rigorous, this procedure does not work for noisy data as the noise tends to be amplified. In many respects, the problem at hand is reminiscent of image deconvolution where images often suffer from noise and the point-spread function (the equivalent of the resolution function) is known. Several algorithms are routinely used in image processing. One is the Wiener deconvolution, where some filtering is carried out in the frequency domain on the Fourier transform to reduce issues of noise amplification. Others, like the steepest descent and Richardson–Lucy algorithms, do work in the time domain and use iterative approaches. We have tested several image deconvolution algorithms available in *Mathematica* (damped least-squares, Tikhonov regularization, truncated singular-value decomposition, Wiener deconvolution, Tikhonov–Golub–Kahan bidiagonalization regularization, Richardson–Lucy, modified residual norm steepest descent). The criteria for judgment were the degree to which the elastic peak approached a Dirac delta function, the lack of deconvolution artifacts near the elastic peak, the accuracy of derived PDOS, and minimal noise amplification. The Richardson–Lucy (Richardson, 1972 ▸; Lucy, 1974 ▸) and steepest descent (Nagy & Strakos, 2000 ▸) methods yielded the best results and the steep­est descent method was adopted for use in *SciPhon*. It is a simple and well established iterative optimization method whose principles are briefly explained below.

Deconvolution of discrete data without noise corresponds to the solution of a system of linear equations. Such a solution can be achieved using iterative methods whereby a starting solution vector is used, which is updated at each iteration using a restoration vector. The restoration vector is calculated so that the discrepancy between the measured spectrum and the solution vector is most rapidly minimized (hence the name steepest descent). The restoration vector can be modified so that no spurious negative counts can be present in the solution vector and noise amplification is limited. Furthermore, to limit noise amplification, the user can limit the number of iterations so that a partial solution is retained while keeping noise amplification within an acceptable range. The iterative solution is appealing because it stands between no deconvolution (no noise amplification but the presence of resolution artifacts) and full deconvolution (significant noise amplification but reduction in resolution artifacts).

**Figure 4:**
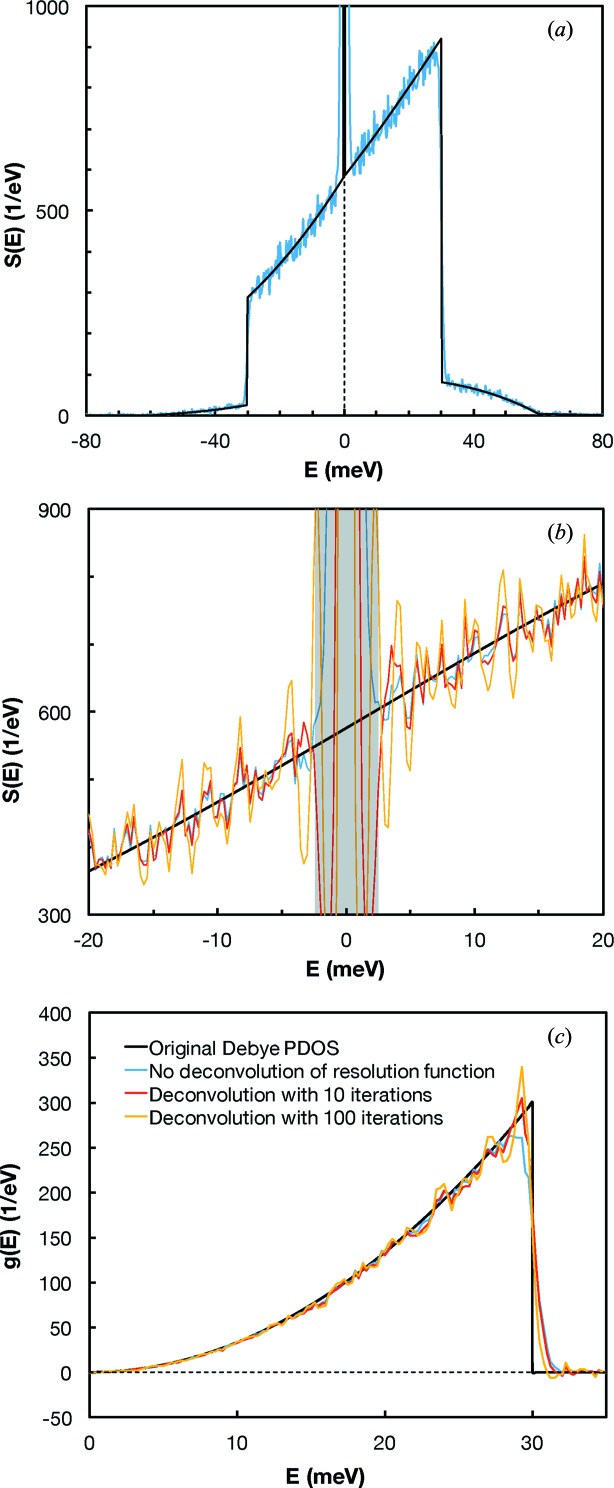
Effect of deconvolution of the resolution function on NRIXS spectra and phonon density of states. (*a*) Synthetic NRIXS spectrum calculated from a Debye PDOS with an energy cutoff of 30 meV (black curve) that was convoluted with a Gaussian-shaped resolution function (FWHM of 2 meV) and to which Poisson noise was added (blue curve). (*b*) Close-up of the NRIXS spectrum near the elastic peak after no deconvolution (blue curve) and deconvolution using the steepest descent with 10 (red curve) and 100 (orange curve) iterations. (*c*) Calculated PDOS from the synthetic NRIXS spectrum with or without deconvolution compared with the input Debye PDOS. A deconvolution using the steepest descent method and 10 iterations is a good trade off between accuracy (the peak in the PDOS at 30 meV is better reproduced) and noise amplification (see text for details).

To test the steepest descent deconvolution technique, we used a synthetic Debye DOS. The rationale for using such a DOS is that it presents a sharp decrease at the Debye energy so the strengths and virtues of the method should be exacerbated. To run the test, we computed a synthetic Debye DOS with a Debye energy cutoff of 30 meV (Fig. 4 ▸). A synthetic spectrum *S*(*E*) was then calculated from this DOS. In NRIXS, the X-rays from nuclear resonant elastic scattering are suppressed relative to those from nuclear resonant inelastic scattering. The elastic peak in the synthetic spectrum was therefore arbitrarily reduced by a factor of ten compared with the rest of the spectrum while in reality the reduction is higher (Mooney *et al.*, 1992 ▸). The synthetic spectrum with suppressed elastic peak was convolved with a synthetic resolution function of Gaussian shape and FWHM of 2 meV. The overall spectrum was rescaled to yield 6000 total counts on the elastic peak. Noise was added to this spectrum using a Poisson distribution. The synthetic spectrum thus produced has many of the features of real NRIXS data (Fig. 4*a* ▸). We then used this synthetic spectrum and associated resolution function as inputs in the *SciPhon* software to calculate the PDOS and all the atomic dynamics and thermodynamic properties that are derived from *g*(*E*) and *S*(*E*). We compared the results following (1) no deconvolution of the resolution function from the data, (2) deconvolution with 10 iterations, and (3) deconvolution with 100 iterations. Near the elastic peak, significant oscillations are present in the deconvoluted data, especially when 100 iterations are performed (Fig. 4*b* ▸). As is expected, the calculated PDOS after 10 or 100 iterations define sharper drops at the Debye energy than when no deconvolution is performed (Fig. 4*c* ▸). However, the deconvoluted data with either 10 or 100 iterations yield noisier PDOS. All the properties derived from the data (deconvoluted or not) are consistent within error bars. As an example, the expected true force constant for a Debye spectrum with a Debye energy of 30 meV is 117.8 N m^−1^. The force constant without deconvolution is 120.2 ± 6.3 N m^−1^, the one after 10 iterations is 119.0 ± 5.5 N m^−1^, and that after 100 iterations is 118.5 ± 5.6 N m^−1^. To summarize, this analysis shows that deconvolution of the resolution function is largely innocuous but can slightly improve the accuracy and reveal subtle features in the PDOS. *SciPhon* leaves to the user the choice of using or not the deconvolution option and, if chosen, of deciding on the number of iterations to perform. Our experience dealing with hundreds of NRIXS spectra is that deconvolution using the steepest descent approach with 10 iterations yields acceptable results (this is the default option in *SciPhon*). Most importantly, it does not add any bias.

### 2.5. Input of background counts and experiment temperature   

The background can be measured away from the resonance by setting the energy to −200 meV relative to the resonance energy, where no counts (even from multiple phonon annihilation) should be present and all the signal should come from the background. This background can be measured for a set duration and the resulting counts can be subtracted from the signal. As discussed below, another option is given to the user, which is to apply a baseline subtraction based on the counts measured in the low- and high-energy tails of the spectrum. In the following, we will refer to background for the counts measured at a single energy far from resonance, and baseline for the counts interpolated from the low- and high-energy tails of the spectrum.

The user is also asked to enter the experiment temperature, meaning the temperature of the sample as it is being measured. This is used when calculating the phonon annihilation part of the NRIXS spectrum from the phonon creation part. Indeed, an option is given in the software to either use the temperature from the detailed balance or that entered by the user. This is particularly useful for experiments carried out at high temperature, where the temperature given by the detailed balance is very imprecise. The input temperature is also used for elastic peak removal (see the section below).

### 2.6. Elastic peak removal   

**Figure 5:**
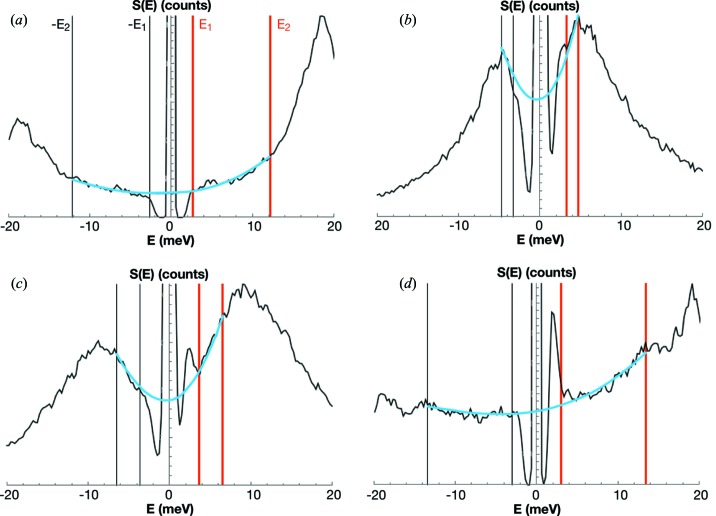
Examples of removal of the elastic peak from the phonon excitation probability density *S*(*E*) by interpolation of the signal at the left and right of the elastic peak. (*a*) Olivine (Dauphas *et al.*, 2014 ▸). (*b*) Fe^3+^-rich rhyolite glass (Dauphas *et al.*, 2014 ▸). (*c*) Fe^2+^-rich basalt glass (Dauphas *et al.*, 2014 ▸). (*d*) Goethite (Dauphas *et al.*, 2012 ▸; Blanchard *et al.*, 2014 ▸). The user moves the red sliders to define the energy range used for the interpolation (red and black vertical markers *E*_1_, *E*_2_, −*E*_1_, −*E*_2_). The blue curve shows the interpolated function using equation (4). In some cases (most minerals) the interpolation is straighforward but in others (*e.g.* glasses) the range available to define the interpolation is narrow. The spectrum between −*E*_1_ and *E*_1_ (elastic peak) is replaced by the interpolated function before further processing. The values of *S*(*E*) near zero, at or near the elastic peak, are artifacts from the deconvolution algorithm (the black curve is the spectrum after deconvolution of the resolution function).

The procedure used by *PHOENIX* for removal of the elastic peak is to rescale the resolution function and to fit it to the undeconvoluted elastic peak. The procedure used in *SciPhon* is different. At two given energies −*E* and +*E*, the NRIXS signal must respect the detailed balance
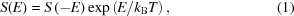
where 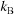 is the Boltzmann constant and *T* is the temperature in K. This is the standard way of writing the detailed balance but it can also be written in the form
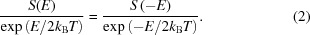
The function 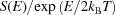 is thus an even function, which can be expanded in a Taylor series as
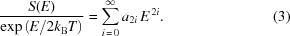
In *SciPhon*, the series is truncated at the second order and near the elastic peak we use the approximation
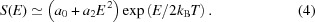
This function is used to extrapolate the NRIXS signal below the elastic peak (Fig. 5 ▸). To do this, the user can move two sliders (denoted 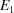 and 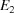 hereafter) that define an energy interval where the data will be used to define the interpolation. The 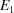 marker is positioned by the user immediately to the right of the elastic peak while the second marker is positioned further away in a range where 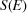 is well fit by equation (4). The parameters 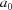 and 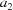 are calculated by fitting equation (4) to 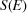 in the interval 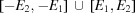. The data 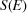 and the fit 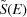 are displayed on the screen in real time as the user adjusts the sliders so that one can directly assess the quality of the fit. In the range 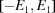, which corresponds to the footprint of the elastic peak, the data points are replaced by the fit function.

### 2.7. Energy truncation and baseline definition   

Removal of a constant background is sometimes not adequate in NRIXS. This issue is most clearly seen in measurements performed over a broad energy range. Beyond a certain energy that depends on the material analyzed, no signal should be detectable above background. In reality, significant counts often remain at low and high energies that cannot be accounted for by multiple phonons. Those counts often do not average to the same values on the low- and high-energy ends of the spectra. As a result, some of the quantities derived from 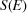 never converge, yielding unreproducible results (Dauphas *et al.*, 2012 ▸, 2014 ▸; Blanchard *et al.*, 2015 ▸; Shahar *et al.*, 2016 ▸). This issue had not been identified before because most studies in NRIXS spectroscopy have focused on the part of the spectrum that is near the elastic peak, where this issue of non-constant baseline is largely inconsequential. However, parameters that depend on accurate measurement of the high- and low-energy ends of the spectrum, such as the mean force constant of iron bonds, are severely affected by this issue of baseline subtraction. To remediate this problem, Dauphas *et al.* (2014 ▸) used a routine for baseline subtraction that relies on acquisition of broad energy scans, the tails of which are used to define a baseline that is subtracted by linearly interpolating the signal between the low- and high-energy tails. The *SciPhon* software gives the option of doing this in an interactive manner using a GUI. A panel displays a zoomed view of the low-energy tail over the interval 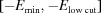, while another panel displays a zoomed view of the high-energy tail between 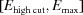 (the total energy acquisition range is from 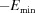 to 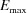). The user can move two sliders to define the energy range beyond which no signal is present (*i.e.* beyond which the signal stops decreasing). The signal in the tails thus defined is cut out and a baseline defined by linear interpolation between the cut out sections is removed from the rest of the spectrum. The user has the option to bypass this truncation/baseline subtraction routine or to manually modify the baseline values to test the sensitivity of the results to baseline subtraction. This routine significantly improves the reproducibility of force constants measured on the same minerals analyzed in different sessions separated by several months or years.

### 2.8. Temperature calculation   

The temperature of the sample can be calculated in NRIXS by using the detailed balance (Lin *et al.*, 2004 ▸)
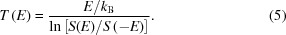
Temperature values can be calculated for every pair of energies −*E* and *E*. For example, for a scan from −120 to +130 meV measured in steps of 0.25 meV, a total of 480 (120/0.25) temperatures can be estimated. *SciPhon* calculates those temperatures and displays them on the screen, so that the user can get a sense of the reliability of the temperature estimate. Not all temperatures have the same error because the counts vary from one energy bin to another. Assuming Poisson statistics, the uncertainty on 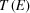 is
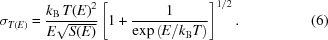
The temperature is calculated by forming the weighted average of the temperatures calculated in each energy bin,
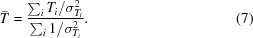
The temperature cannot be reliably estimated from the detailed balance for high-temperature experiments because the relative error increases with temperature: 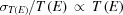. After calculating the temperature from the detailed balance, the user is given the choice to either use this temperature or the one entered (§2.5). The temperature thus chosen (either calculated from the detailed balance or provided as input from the user) is used subsequently in calculation of the DOS and other parameters.

### 2.9. Normalization of *S*(*E*), calculation of the Lamb–Mössbauer factor, the DOS and extrapolation of *S*(*E*) beyond the truncation range   

Calculation of the PDOS from a NRIXS spectrum was first performed by Sturhahn *et al.* (1995 ▸) (also see Sturhahn, 2000 ▸, for details). The first step in the calculation of the phonon density of states 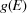 is the normalization of the nuclear resonant spectrum 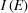 using Lipkin’s sum rule (Lipkin, 1962 ▸, 1995 ▸, 1999 ▸). It stipulates that the first moment of the excitation probability density must be equal to the recoil energy,
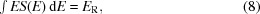
where 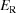 = 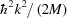 = 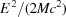 = 1.96 meV for 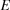 = 14.4125 keV (*k* is the wavenumber, *M* is the mass of ^57^Fe, *c* is the speed of light and 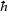 is the reduced Planck’s constant). 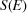 comprises an inelastic term 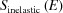 and an elastic term 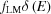, where 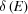 is the Dirac delta function and 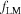 is the Lamb–Mössbauer factor. Because the Dirac delta function is even, it cancels out in the integral and 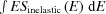 = 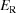. The procedure described in §2.6 provides a means of removing the elastic peak and retrieving the inelastic part of the phonon excitation probability density 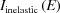, which is proportional to 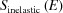,
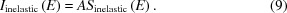
Using equation (8), we can calculate the normalization factor,
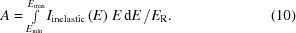
This factor is used to rescale the intensity spectrum, which has units of counts, into an excitation probability density 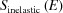, which has units of inverse of the energy (1/eV). Note that the measured phonon excitation probability density is a convolution of the density 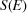 and the resolution function 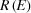. As discussed in §2.4, the first step in the procedure is to deconvolve the resolution function from the spectrum. If this was not done and the resolution function had a non-zero first moment, a correction would need to be applied to the normalization factor: 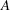 = 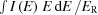 − 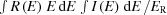 (Sturhahn *et al.*, 1995 ▸). Similar corrections would also be needed for the higher-order moments (Hu *et al.*, 2013 ▸). However, these are unnecessary in our data reduction procedure because the normalization and all subsequent data treatment is performed on a deconvolved spectrum.

One can then calculate the Lamb–Mössbauer factor 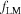 using the zeroth moment of 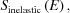


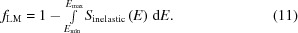
The full excitation probability density, including the elastic peak, can be reconstructed by adding a Dirac delta function of integral 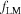,
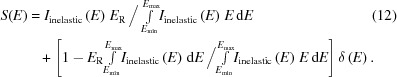
As explained below, the normalized function 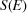 is used to calculate the PDOS.

Assuming that the lattice is harmonic, meaning that the potentials vary as the square of the atomic displacements, it is possible to calculate the DOS 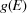 from 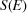. We have the following expressions,
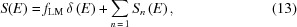


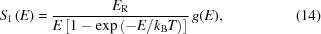


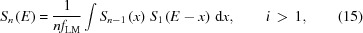
where 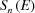 is the *n*-phonon contribution. The measured spectrum 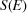 after normalization is thus a combination of 1, 2,…, *n* contibutions and each *n* term is the convolution of the 1 phonon and 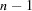 phonon contributions. Kohn *et al.* (1998 ▸) and Sturhahn (2000 ▸) showed, using formulas previously derived in the context of electron scattering (Johnson & Spence, 1974 ▸), that the 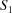 phonon contribution could be derived from *S* using the Fourier–Log method. Because the Fourier transform of two convoluted functions is the product of their Fourier transforms, the Fourier transform of equation (15) yields
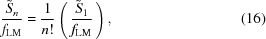
where 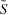 is the Fourier transform of 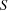. The Fourier transform of equation (13) that gives the full excitation probability density is
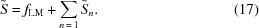
Combining those two equations, we have
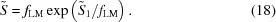
The single-phonon contribution can therefore be calculated from the excitation probability density using the formula
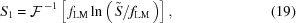
where 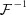 is the inverse Fourier transform. Once 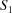 is known, it is straighforward to calculate the partial (only reflecting iron excitation) projected (along the measurement direction) phonon density of states,
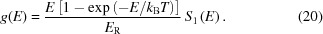
The phonon annihilation and creation parts of the spectrum both convey some information on the PDOS as 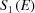 is related to 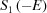 through the detailed balance. Let us denote 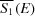 as the weighted average value calculated from 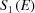 and 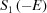. We assume that the errors in 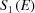 and 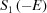 scale as the square-root of those values. It follows that the average weighted by the inverse of the variance is
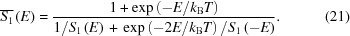
We therefore have, for 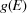,
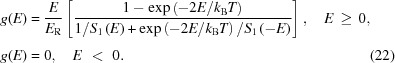

*SciPhon* calculates 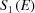 using the fast Fourier transform routine implemented in *Mathematica*. Once the single-phonon contribution has been calculated, 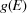 is computed using equation (22).

A potential difficulty with NRIXS is that the signal at high energy can influence the calculated parameters even when the signal is near or below the baseline. To mitigate this issue, *SciPhon* recalculates 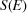 from 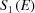 at energies that are beyond the energy acquisition or truncation ranges. This is equivalent to the ‘refinement’ method used in *PHOENIX* and it allows extrapolation of 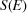 outside the truncation/acquisition range in a physically sound manner. Once this extrapolation is done, the synthetic part of the spectrum is appended to the measured spectrum and this new ‘augmented’ spectrum is used to recalculate 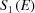 and 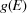. This procedure is repeated twice, ensuring convergence and consistency of the parameters derived from 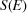 and 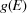.

### 2.10. Calculate sound velocities   

An important use of NRIXS in geophysics and high-pressure mineral physics is the determination of seismic velocities at high pressure–temperature (Mao *et al.*, 2001 ▸; Hu *et al.*, 2003 ▸). The shear (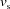) and compressional (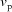) velocities are not measured directly in NRIXS. Instead, one can measure the Debye velocity (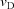). This velocity is calculated from the NRIXS spectrum near the elastic peak. While some quantities derived from NRIXS measurements depend dramatically on removal of the background/baseline, determination of the Debye velocity depends on achieving a good resolution so that the contribution from the elastic peak is small. For most solids, the spectrum near the elastic peak shows Debye-like behavior, meaning that the PDOS increases quadratically with the energy (glasses can show departure from this behavior in the form of a Boson peak; Chumakov *et al.*, 2011 ▸),
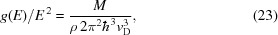
where *M* is the mass of the nuclear resonant isotope and ρ is the density. In *SciPhon*, the user is asked to enter the density ρ and bulk modulus *K* of the material being examined. The function 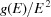 is plotted as a function of *E*. For a pure Debye behavior, it should be constant over some interval. In practice, this is not often achieved, partly because the low-energy range of the spectrum is below the elastic peak. To address this difficulty, the function 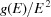 is fitted by a low-degree series expansion with a derivative that is zero at the origin,
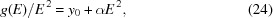
where 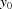 is the intercept at 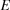 = 0 and it has unit of 1/energy^3^. The user can move two vertical sliders that define the energy range over which the data are fitted by this function. A black vertical marker indicates the energy that the user has previously defined to remove the elastic peak from the spectrum by extrapolation (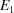 in §2.5). The data points below this energy are not real data as they are derived from interpolated values during elastic peak removal, and the user is advised against using them in the fit. Nevertheless, they provide another test to assess the robustness of the fit and the Debye velocity estimate. Indeed, it is possible to compare the intercept (*a*) calculated by fitting the function 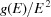 using equation (24) with the intercept given by processing the interpolated 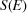 through the whole procedure for deriving 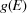. Both interpolations are series expansions but they are performed in different spaces so agreement between the intercepts obtained using both approaches gives confidence that the Debye velocity estimate is reliable. Knowing 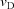, ρ and *K*, it is possible to calculate 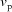 and 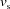 by solving the following system (Mao *et al.*, 2001 ▸),
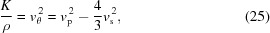


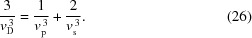
These formulae are strictly valid for isotropic media only, and are not valid for crystals, including cubic ones or powders (Bosak *et al.*, 2016 ▸). To propagate the uncertainties from 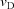, *K* and ρ, we use approximate solutions to these equations,
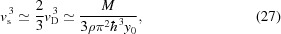


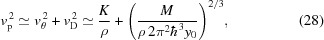
with 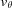 = 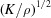. It follows that
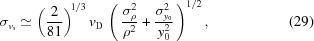


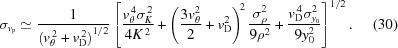
The Poisson ratio that relates transverse to axial strain can be calculated from 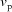 and 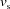 using the formula
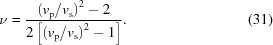

*SciPhon* reports this value, which expectedly is always close to 0.3 for metals.

## 3. Derived parameters   

In addition to temperature, many parameters can be derived from the excitation probability distribution 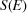 and from the PDOS 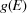 (Sturhahn, 2000 ▸; Kohn & Chumakov, 2000 ▸; Dauphas *et al.*, 2012 ▸; Hu *et al.*, 2013 ▸). Some parameters can be derived from both functions, and one should always make sure that the parameters are in agreement. Below, we give the formulas used in *SciPhon* to calculate those parameters. Many of these formulas depend on the moments of *S* and *g*, so we use the following notations,
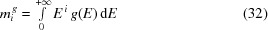
for the *i*th moment of the PDOS,
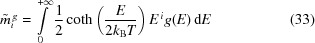
for the *i*th thermalized moment of the PDOS,
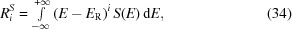
for the *i*th central moment of the excitation density distribution.

### 3.1. Parameters from *S*   

*Temperature *T*.* This has already been discussed at length in §2.8 and will not be repeated here. The detailed balance equation is used to calculate *T* through [equation (5)]
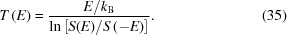



*Lamb–Mossbauer factor* 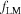. The Lamb–Mössbauer factor is the ratio of recoil-free to total nuclear resonant absorption. It follows from the normalization of the phonon excitation probability function [equation (11)],
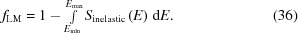



*Mean-square displacement* 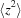. The mean-square displacement of atoms in their potential is related to the Lamb–Mössbauer factor through
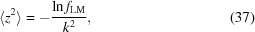
where 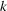 is the wavenumber of the photons with 14.4125 keV energy [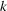 = 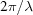 = 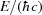 = 7.30 Å^−1^]. Note that the wavenumber *k* should not be mistaken for the Boltzmann constant 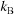.

*Kinetic energy per atom KE*. This is the energy associated with atomic motions along the direction of the X-ray beam,
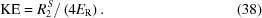



*Internal energy per atom *U**. This is the total energy of the iron sublattice and it comprises kinetic energy from atomic motions and potential energy from chemical bonds. For a harmonic oscillator, the internal energy is equally partitioned between kinetic and potential energy, so we have
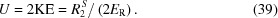



*Mean force constant 〈*F*〉*. The third central moment of 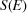 gives the second derivative of the potential, which for a harmonic oscillator is the force constant,
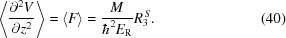
Dauphas *et al.* (2012 ▸) compiled mean force constant determinations for iron-bearing compounds published until 2012.

*Isotopic fractionation factors β*. At equilibrium, the various isotopes of an element are not distributed randomly between coexisting phases. Instead, heavy isotopes tend to partition into phases that form stronger bonds (Bigeleisen & Mayer, 1947 ▸; Urey, 1947 ▸). This is quantified using reduced partition function ratios, or β-factors, which correspond to equilibrium isotopic fractionation factors between the phase of interest and monoatomic gas of the same element. If this β factor is known, then it is straightforward to calculate equilibrium fractionation between coexisting phases. We can write an isotope exchange reaction for ^54^Fe and ^56^Fe between solid-phase Fe*X* and monoatomic gaseous Fe,
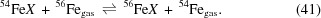
The equilibrium constant for this reaction (substituting isotopes form near ideal solutions) is
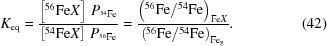
This is the expression of the β-factor or reduced partition function ratio used in isotope geochemistry. Because isotopic variations are small, 1000lnβ is more often reported than the absolute value of β. Dauphas *et al.* (2012 ▸) and Hu *et al.* (2013 ▸) established relationships between the even moments of *g*(*E*) and the moments of *S*(*E*) to calculate the β-factors at any temperature as a function of the moments of *S*(*E*),
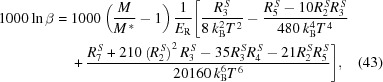
where *M* and 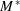 are the masses of the two isotopes. This can be rewritten as 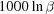 = 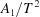 + 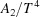 + 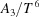, where the coefficients 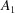, 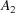 and 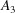 can be readily identified with the terms in equation (43). *SciPhon* calculates and reports all these coefficients. Truncating the formula above, we obtain the approximate formula
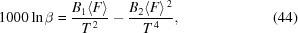
where 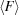 is the mean force constant of the iron bonds [equation (40)]. 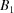 is fixed for a given element (2904 for iron) while 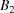 depends on the phase that is being considered. For a Debye PDOS and for iron, 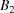 = 37538. Most phases have a PDOS that corresponds to a 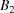 value of ∼52000 for iron. *SciPhon* reports the value of 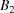 calculated based on equation (43).

### 3.2. Parameters from *g*   

*Mean square displacement and Lamb–Mössbauer factor*. The atoms oscillate in their potential around an equilibrium position. The magnitude of these oscillations is quantified by the mean square displacement (*i.e.* the mean value of the square of the displacement). This can be calculated from a thermalized moment of the PDOS,
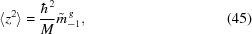
where *M* is the mass of the nuclear resonant isotope. The Lamb–Mössbauer factor is calculated from the mean square displacement through [equation (37)]
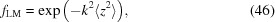
where 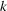 is the wavenumber. The temperature-dependence of the mean square displacement is given by
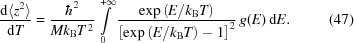
At high temperature when 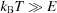 over most of the phonon spectrum (the temperature corresponding to a Debye energy of 30 meV is 350 K), this can be approximated by 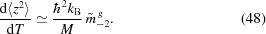
For energies close to zero, the integral is not well defined but we can use the fact that near zero we have
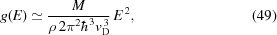


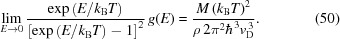
The *SciPhon* program calculates the Debye velocity [*i.e.* the proportionality constant between 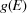 and 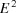]. The integral giving 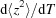 in equation (47) is thus split into two domains, one where the term under the integral is given by equation (50) (the limit in energy corresponds to the upper bound of the elastic peak as defined by the user) and another where the full formula [equation (47)] is used.

*Critical temperature.* The Lamb–Mössbauer factor decreases when the temperature increases. The temperature increment corresponding approximately to a factor of 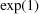 = 2.7 decrease in 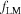 is called the critical temperature and is calculated as
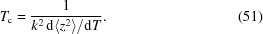



*Resilience*. Another quantity related to the mean square displacement is the resilience, introduced in the study of protein dynamics (Zaccai, 2000 ▸; Leu & Sage, 2016 ▸). It has the same unit as the force constant (N m^−1^) and its expression is
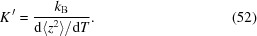



*Internal and kinetic energy*. The partial and projected internal energy (kinetic and potential) is given by
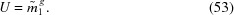
The Virial theorem says that the internal energy must be equally partitioned between potential and kinetic energy. The kinetic energy associated with vibrations along the measurement direction is thus given by
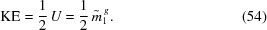



*Vibrational entropy*. Entropy can take two forms in solids: vibrational and configurational. The latter corresponds to atomic disordering when several non-identical atoms can occupy the same site as is the case for solid solutions. The former corresponds to the thermal agitation of atoms around their equilibrium positions. Its expression is 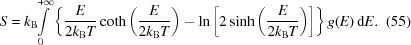



*Helmoltz free energy*. The projected partial Helmoltz free energy is given by the expression 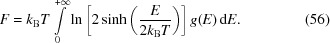



*Lamb–Mössbauer factor and kinetic energy at *T* = 0 K*. At 0 K, solids still possess quantum mechanical zero-point energy. The kinetic energy at 0 K can be calculated from NRIXS spectra using the formula KE = 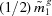, where a temperature of 0 K is used in the expression of 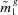. Similarly, the Lamb–Mössbauer factor at 0 K can be calculated using 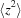 = 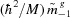 adopting *T* = 0 K in 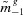.

*Mean force constant*. The mean force constant of the bonds holding the resonant isotope in position is given by the second moment of the PDOS, which is mathematically related to the third moment of 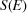,
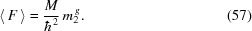



*Isotopic fractionation factors β*. The isotopic fractionation factors, or more specifically the coefficients in the temperature expansion of 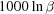, can be calculated from the kinetic energy or the even moments of 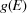. The relationship was established by Polyakov *et al.* (2005 ▸) using the kinetic energy and first-order perturbation theory while Dauphas *et al.* (2012 ▸) derived the formula using a Bernoulli expansion of the reduced partition function ratio. In the expression 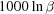 = 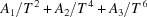, the coefficients can be identified with the formula below,
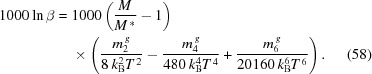



*Comparison of the isotopic fractionation factors β at the experiment temperature.* The value of 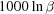 can be calculated from both 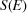 and 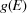 at any temperature, including the experiment temperature, using the polynomial’s expansions in even powers of the inverse of the temperature [equations (43) and (58)]. Polyakov *et al.* (2005 ▸) also give a formula that gives 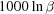 as a function of the partial kinetic energy, whose value can be calculated using both 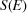 and 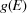. The expression for 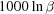 is
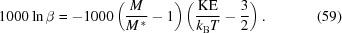

*SciPhon* calculates and outputs the value of 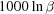 at the experiment temperature using these four approaches (polynomial expansion or kinetic energy using *S* or *g*), which are mathematically equivalent. This allows the user to assess the consistency of those calculated values. In our experience, the values are always consistent to within a few percent.

### 3.3. Calculation of error bars on derived parameters   

Calculating the errors on the parameters derived from 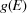 is not straightforward because the errors of the PDOS at different energies are correlated. Indeed, the value of *g* at any given energy *E* depends on the values of 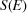 at all energies due to the various normalizations and Fourier–Log transform involved. In the present implementation of *SciPhon*, the errors are not propagated in the parameters derived from 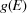. We plan to implement this capability in a future version of *SciPhon*, presumably using a Monte Carlo approach.

The errors on the parameters derived from 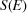 are more straightforward to compute. Details on the error propagation calculation are provided by Hu *et al.* (2013 ▸), Dauphas *et al.* (2014 ▸) and Hu (2016 ▸). The uncertainties that are propagated are presented below. Note that all sources of error are not well quantified and the default values adopted below are conservative. The user can easily change these uncertainties in *SciPhon*.

(1) Counting statistics that follow a Poisson distribution.

(2) Uncertainties in the baseline definition, which is given by the uncertainty in the interpolation between the truncated low- and high-energy ends.

(3) Offset in energy scaling. The resonance energy is found automatically by the padd routine of the *PHOENIX* package by finding the maximum in 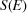 corresponding to the elastic peak. The energy resolution of the scans is ∼1 meV but the scans are made with energy steps much smaller than that (typically 0.25 meV). In a single scan, the maximum intensity in the spectrum, which sets zero in the energy scale, is not known to better than half of the energy step size (0.25/2 ≃ 0.1 meV). Several energy scans are stacked and the energy scale zero is known with a better precision than 0.1 meV. As a conservative approach, we adopt a default uncertainty of 0.1 meV in the possible offset in the energy scale relative to the resonance energy but the user can set it to a lower value if so desired.

(4) Overall energy scaling. If the energy scale is not perfectly calibrated, this could result in stretching or compression of the energy scale relative to the true value. The absolute energy scaling is checked at the beginning of each session by measuring an iron foil characterized by a sharp decrease in the PDOS at an energy of 35–40 meV. Therefore, the effect is probably minor. Nevertheless, we have adopted a default uncertainty of 1% of this energy scaling.

(5) Bin-to-bin energy jitter. The scans in energy are performed in increments by the motion of crystals in the high-resolution monochromator. This can induce some bin-to-bin jitter in energy. A default jitter value of 0.1 meV is used. The net effect of this source of uncertainty is small compared with others because this jitter largely cancels out for the large number of energy increments used during a scan.

All these sources of error are combined quadratically into an overall error for the derived parameters.

## 4. Discussion   

The software most often used for reducing NRIXS data at sector 3ID of the APS is *PHOENIX* (Sturhahn, 2000 ▸). The *SciPhon* software presented here presents several distinguishing features. Most notably, (1) it is a GUI software with interactive sliders and fill spaces that streamlines some tasks associated with the data reduction (Fig. 3 ▸), (2) it outputs all the parameters needed to calculate reduced partition function ratios that are used to predict equilibrium isotopic fractionation between coexisting phases in geochemistry, (3) it uses a different approach to remove the elastic peak based on an interpolation of the signal near the elastic peak, (4) it offers the option of running an iterative peak deconvolution algorithm, (5) it offers the option of truncating the energy range used in the data analysis, and (6) it streamlines the definition and removal of a non-constant baseline across the energy range, which is important for the determination of the parameters derived from the higher moments of 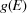 and 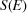. Below, we highlight how some of these differences affect the results and assess the quality of the data reduction algorithm.

### 4.1. Deconvolution of the resolution function and spectrum   

**Figure 6:**
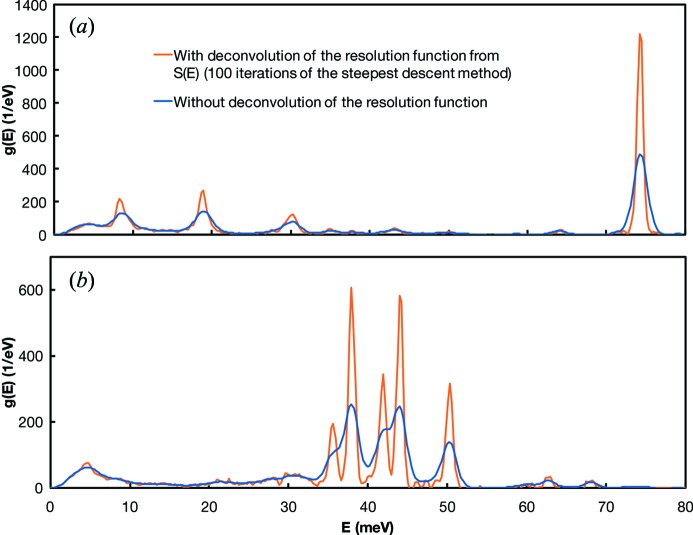
Effect of the deconvolution algorithm on the PDOS of nitro­syl iron porphyrinate derivative (heme)[Fe(oep)NO] for NRIXS spectra measured out-of-plane [(*a*) OP; perpendicular to the porphyrin plane] and in-plane [(*b*) IP; parallel to the porphyrin plane] (Pavlik *et al.*, 2010 ▸). The blue curves are undeconvoluted data while the orange curves are the PDOS calculated from *S*(*E*) after deconvolution of the resolution function using the steepest descent method and 100 iterations. As shown in (*b*), peaks that would be unresolvable are clearly separated after deconvolution of the resolution function.

As discussed in §2.4, the deconvolution algorithm has little influence on the calculated results. This includes the estimation of the Debye velocity, which is close to the elastic peak and could potentially benefit or suffer from the deconvolution algorithm. Where deconvolution provides the most benefit is in sharpening the peaks and improving the resolution of the spectrum. The downside of the deconvolution is that it amplifies the noise but this is not really an issue where signal is well above background. Where the deconvolution algorithm is most useful is in biochemistry, where the NRIXS technique (also known as NRVS) is used to study the vibrational properties of iron-bearing biomolecules (Sage *et al.*, 2001 ▸; Scheidt *et al.*, 2005 ▸; Rai *et al.*, 2002*a* ▸,*b* ▸, 2003 ▸; Leu *et al.*, 2007 ▸, 2008 ▸; Lehnert *et al.*, 2010 ▸). In those biomolecules, very specific vibration modes dominate and the PDOS often features well defined peaks. The peak positions in the PDOS can be compared with theoretical models to test hypotheses on molecular configurations. To assess the usefulness of the deconvolution algorithm, we have performed the data reduction on previously acquired NRVS spectra of nitro­syl iron porphyrinate derivatives (Pavlik *et al.*, 2010 ▸). These spectra are ideally suited to test the algorithm because the non-deconvoluted spectrum shows overlapping adjacent peaks that cannot be fully resolved. The result of the deconvolution is shown in Fig. 6 ▸. The peaks that could not be resolved prior to deconvolution can be clearly distinguished when the resolution function is deconvoluted from 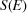. The iterative steepest descent algorithm is thus appropriate for applications of NRVS to biochemistry.

### 4.2. Round-robin test   

We routinely compare the results from *SciPhon* with those from *PHOENIX* and we have found excellent agreement when the exact same procedure is used. For example, *PHOENIX* does not offer any built-in feature to remove a non-constant baseline but this can be done offline on the data file so that a truncated and baseline-corrected file can be used as input in *PHOENIX*.

**Figure 7:**
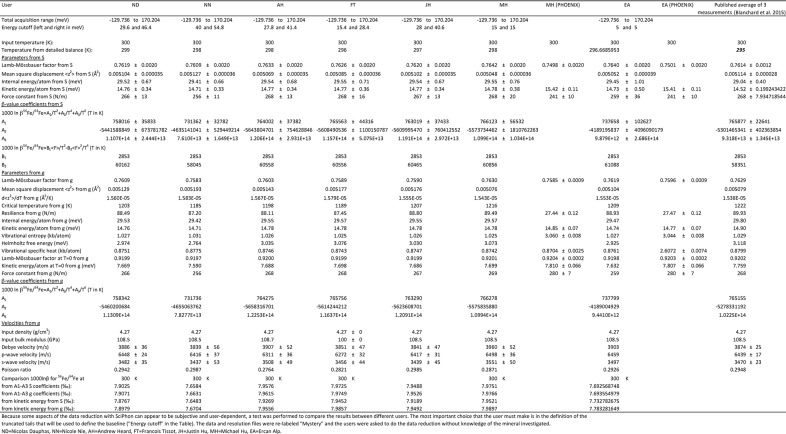
Comparison between users on the data reduction of goethite NRIXS data (*i.e.* ‘Goethite 2’ from Blanchard *et al.*, 2015 ▸).

Our previous results of force constant determinations have shown that the results could be affected by the presence of a non-constant baseline (Dauphas *et al.*, 2012 ▸, 2014 ▸; Roskosz *et al.*, 2015 ▸; Blanchard *et al.*, 2015 ▸). Despite an extensive investigation, we are still unsure as to why the baseline is sometimes not constant between the low- and high-energy tails of the spectrum. The issue is particularly important for the parameters derived from higher-order moments of 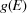 or 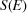 because the signal at high energy is low and close to the background but its contribution to higher-order moments is significant because it is multiplied by a large power of the energy. In *SciPhon*, the user decides visually what part of the spectrum will be used to define the baseline and will be truncated. Similarly, the user is involved in deciding, on the basis of a graph, what the bounds are for the resolution function, what part of 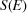 is used to remove the elastic peak by interpolation, and what part of 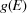 is used to calculate the Debye velocity. There is thus a certain level of subjectivity involved in reducing NRIXS data. A virtue of *SciPhon* is that the relevant graphs are provided to the users to help them make informed choices. In principle, there is no reason why those tasks could not be handled algorithmically but implementing these would be tedious while they can easily be performed by the user. In order to assess the degree to which those user decisions affect the model output, the lead author organized a round-robin test. The files corresponding to some NRIXS measurements of goethite powder (‘Goethite 2’ in Table 1 of Blanchard *et al.*, 2015 ▸) were distributed to several of the co-authors. The files containing the data and resolution were relabeled ‘mystery.dat’ and ‘mystery.res’, respectively, and the participants were never informed of the nature of the phase that they were analyzing. The result of this comparison between seven users is compiled in Fig. 7 ▸. The reasons why goethite was used as a test case are that (i) it is rich in ^57^Fe and good quality data can be acquired in a short time span so we measure it regularly to assess the long-term reproducibility of the technique, and (ii) this particular data set was difficult to handle as the spectrum has tails at the low- and high-energy ends that are not identical and it is not completely clear whether some of the features in those tails belong to the signal or the baseline. As a result, the seven users decided to truncate the data over very different intervals. The data were acquired over an energy range of [−130; +170] (meV). The data ranges defined by the users varied from [−125; 165] (*i.e.* little truncation; user EA in Fig. 7 ▸) to [−90; 115] (*i.e.* extensive truncation of the tails; user NN in Fig. 7 ▸). Despite those disparate choices, the calculated force constant varies little; from 256 ± 11 N m^−1^ to 268 ± 13 N m^−1^ (the force constant is used for comparison purposes because it is highly sensitive to the baseline definition). All other parameters derived from the NRIXS data also agree well, including the Debye velocity. The range of Debye velocities is from 3839 ± 56 m s^−1^ to 3960 ± 52 m s^−1^. All the parameters derived from the data also agree well with the mean goethite values published by Blanchard *et al.* (2015 ▸), which correspond to average values from three independent measurements performed in different sessions over several years. The conclusion of this test is that user choices do not dramatically influence the parameters calculated from NRIXS data and that the user-to-user dispersion is within the quoted errors for most parameters, even for data sets for which data reduction is not straightforward, such as the goethite data used as a test case. We expect the user-to-user dispersion to be smaller in most cases when the NRIXS spectra are better behaved.

This goethite spectrum also provides us with the opportunity to compare the model outputs from *SciPhon* and *PHOENIX*. Co-authors Michael Hu and Ercan Alp, who are well versed in the use of *PHOENIX*, carried out the data reduction on the same sample using *PHOENIX* (Fig. 7 ▸). The calculated force constant is slightly higher with *PHOENIX* [280 N m^−1^ calculated from *g*(*E*) after refinement] than with *SciPhon* but still within error. The difference is most likely due to the fact that a constant background is subtracted with *PHOENIX* while a linearly interpolated baseline is subtracted from the data with *SciPhon*. The most obvious difference between the *SciPhon* and *PHOENIX* outputs are the values of the resilience. *SciPhon* gives a value of ∼88 N m^−1^, while *PHOENIX* gives a value of ∼27 N m^−1^. The critical temperatures in *PHOENIX* and *SciPhon* are very close (∼1200 K). Resilience (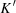) and critical temperature (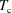) are related through 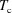 = 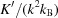. We found internal consistency between these values in *SciPhon* but not in *PHOENIX*. Finally, *PHOENIX* reports errors that are significantly smaller than those reported by *SciPhon*. This is because *PHOENIX* only propagates counting errors, while *SciPhon* propagates those same errors as well as uncertainties associated with the subtraction of a baseline and error in the energy scaling (see §3.3). Our experience is that the counting errors alone cannot explain the session-to-session variability in some parameters, suggesting that the inclusion of other sources of uncertainty in the error propagation scheme is not only justified but necessary.

### 4.3. Application to Mössbauer isotopes other than iron   

*SciPhon* has been used primarily to reduce NRIXS data for iron but it was designed to handle other Mössbauer isotopes. The isotopes provided by default are ^57^Fe, ^119^Sn, ^151^Eu, ^161^Dy and ^83^Kr, which have been measured at the APS. We can easily add other elements upon request by modifying a few lines in the code. The code was extensively tested for iron and several publications have already made use of it (Dauphas *et al.*, 2014 ▸; Roskosz *et al.*, 2015 ▸; Blanchard *et al.*, 2015 ▸; Shahar *et al.*, 2016 ▸; Liu *et al.*, 2017 ▸). Other Mössbauer isotopes may present other challenges to the data reduction algorithm. As a test of how the algorithm performs on non-iron isotopes, we have reduced NRIXS data for SnO (Giefers *et al.*, 2006 ▸), SnO_2_ (Hu *et al.*, 1999 ▸), Kr at 2 GPa (Zhao *et al.*, 2001 ▸), EuO and Eu_2_O_3_ (see supporting information for the values of the PDOS for these compounds).

**Figure 8:**
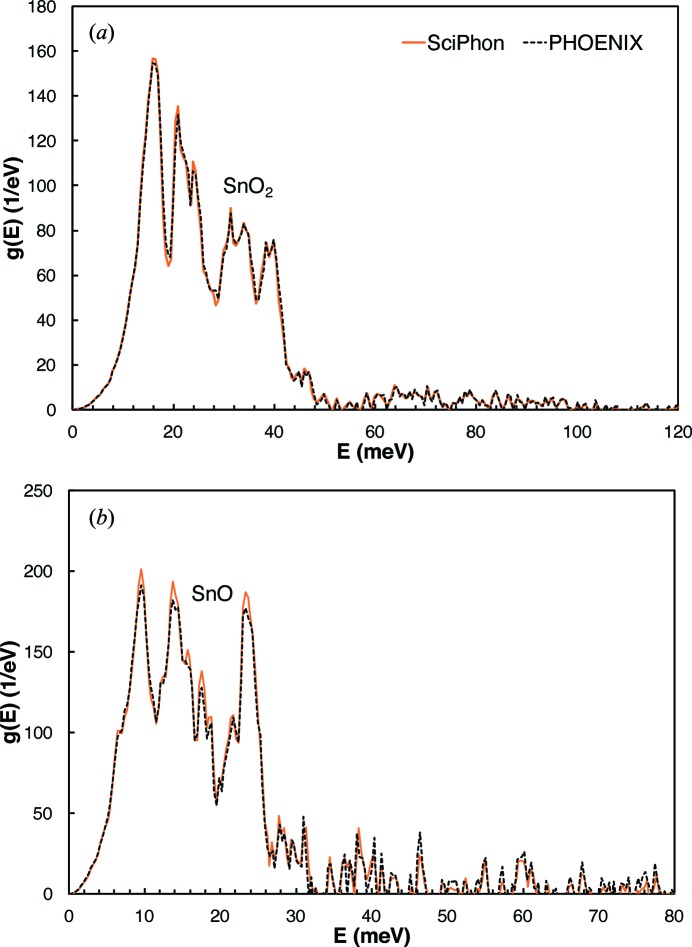
Comparison between the Sn partial phonon density of states of SnO_2_ (cassiterite; Hu *et al.*, 1999 ▸) and SnO (Giefers *et al.*, 2006 ▸) at 300 K calculated using the *PHOENIX* (dashed black curve) and *SciPhon* (solid orange curve). There is excellent agreement between the two, demonstrating that *SciPhon* can handle data reduction of Mössbauer isotopes other than iron.

An important difference between iron and tin is that the Lamb–Mössbauer factor of tin compounds is usually small and multi-phonon contributions are significant (Hu *et al.*, 1999 ▸). Fig. 8 ▸ compares the PDOS of SnO_2_ and SnO calculated by *PHOENIX* and *SciPhon*. As shown, there is excellent agreement between the two. We also examined the parameters calculated by *PHOENIX* and *SciPhon* and there is again excellent agreement between the two. The only major disagreement concerns the resilience, which *SciPhon* calculates at 159 N m^−1^ while *PHOENIX* gives a value of 51 N m^−1^. As discussed above in the context of ^57^Fe NRIXS measurements, the critical temperature estimates agree between *SciPhon* and *PHOENIX* but there is no internal consistency between the critical temperature and resilience calculated by *PHOENIX*. Another difference concerns the vibrational entropy and vibrational specific heat, which differ by a factor of three (the output values of *PHOENIX* are higher than those of *SciPhon*). The values calculated by *SciPhon* are directional, meaning that they would have to be multiplied by a factor of three to account for the bulk material if it was isotropic. This factor of three is not the cause for the discrepancy between the two softwares for the resilience.

**Figure 9:**
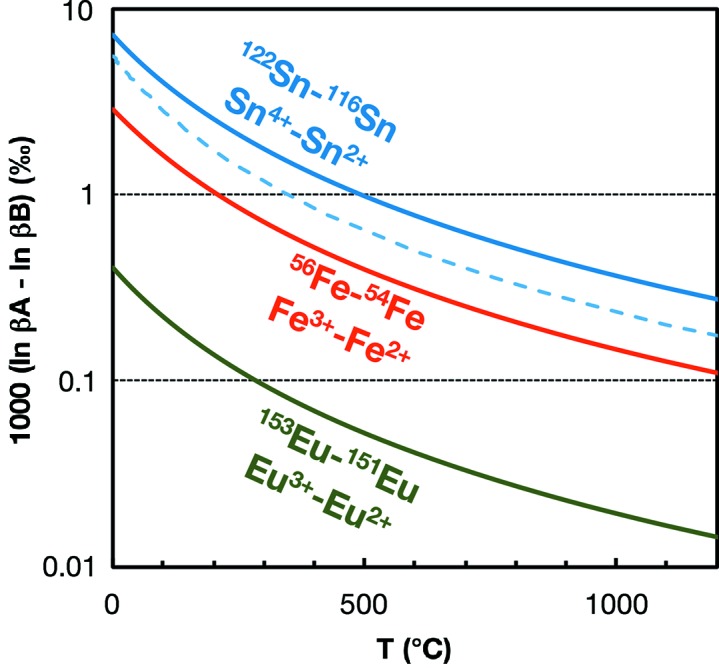
Predicted equilibrium fractionation factors (in ‰) of the redox pairs Sn^4+^/Sn^2+^ (SnO_2_, SnO) (Giefers *et al.*, 2006 ▸; Hu *et al.*, 1999 ▸), Fe^3+^/Fe^2+^ (Fe_2_O_3_, FeO) (Dauphas *et al.*, 2014 ▸) and Eu^3+^/Eu^2+^ (Eu_2_O_3_, EuO); see text for details. The blue dashed line is the previous estimate for the Sn^4+^/Sn^2+^ redox pair by Polyakov *et al.* (2005 ▸). The solid blue line is the new updated value.

**Figure 10:**
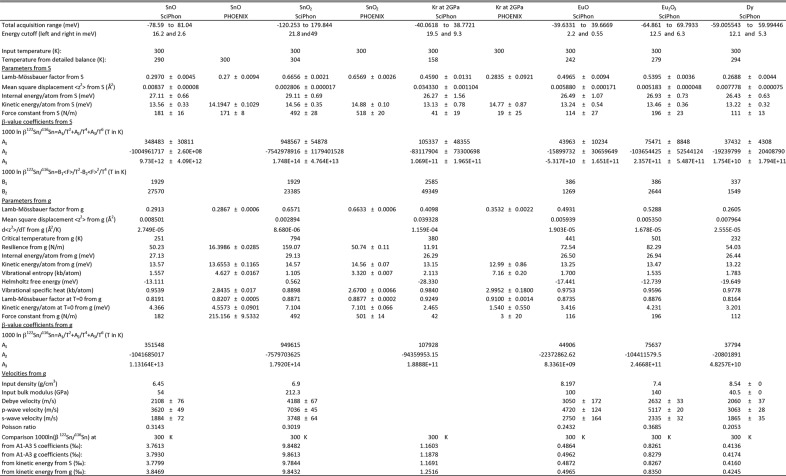
NRIXS data for Sn, Kr, Eu and Dy compounds reduced with the *SciPhon* software (a comparison with *PHOENIX* is provided for Sn and Kr).

Polyakov *et al.* (2005 ▸) used previously published NRIXS data of SnO and SnO_2_ to calculate the β fractionation factors for those compounds. These β-factors give the extent of tin isotopic fractionation between coexisting phases at equilibrium. To calculate those β-factors, they digitized published data. As discussed by Dauphas *et al.* (2014 ▸) and Blanchard *et al.* (2015 ▸), great care must be exercised in handling the low- and high-energy tails of NRIXS spectra for application to isotope geochemistry, so it is worth re-evaluating the fractionation factors for Sn (Fig. 9). The polynomial expansion to the β-factors for Sn are (the temperature *T* is in K), for SnO,
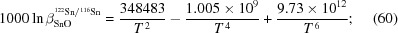
for SnO_2_,
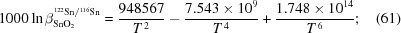
and the predicted equilibrium fractionation between Sn^4+^ (SnO_2_) and Sn^2+^ (SnO) is
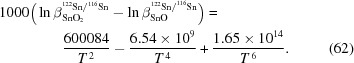
We have also reduced NRIXS data for EuO and Eu_2_O_3_. The polynomial expansion to the β-factors for Eu are (the temperature *T* is in K), for EuO, 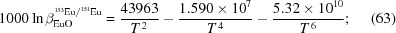
for Eu_2_O_3_,
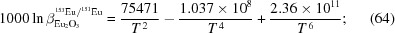
and the predicted equilibrium fractionation between Eu^3+^ (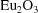) and Eu^2+^ (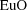) is
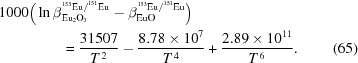
Previous NRIXS studies have investigated the force constants of iron bonds in FeO (wüstite) and Fe_2_O_3_ (haematite) (Dauphas *et al.*, 2017 ▸). The equilibrium isotopic fractionation between Fe^2+^ and Fe^3+^ in oxides has important geologic implications (Dauphas & Rouxel, 2006 ▸; Dauphas *et al.*, 2009*a* ▸, 2017 ▸; Roskosz *et al.*, 2015 ▸), and the redox pairs Sn^4+^/Sn^2+^ and Eu^3+^/Eu^2+^ could similarly provide insights into redox processes in the Earth and other planetary bodies. In Fig. 9 ▸ we plot the equilibrium fractionation factors for all three redox couples (Fig. 10 ▸). The system that shows the largest fractionation is Sn, followed by Fe and then Eu, which reflects in part the large relative mass difference between the isotopes involved (Δ*m*/*m* = 0.05 for ^122^Sn/^116^Sn, 0.036 for ^56^Fe/^54^Fe and 0.013 for ^153^Eu/^151^Eu). At room temperature (25°C), the calculated fractionations are 6.2‰ for Sn, 2.5‰ for Fe and 0.34‰ for Eu. At a temperature of 1100°C that is more relevant to magmatic systems, the calculated fractionations are 0.32‰ for Sn, 0.13‰ for Fe and 0.02‰ for Eu. The isotopic composition of Sn can be measured with a precision of less than ±0.04‰ (Wang *et al.*, 2017 ▸; Creech *et al.*, 2017 ▸) and that of Fe can be measured with a precision of ±0.03‰ (Dauphas *et al.*, 2009*b* ▸, 2017 ▸). There is extensive literature documenting redox-controlled Fe isotopic fractionation in laboratory experiments and natural systems, including low-temperature aqueous systems and high-temperature magmatic systems (Dauphas & Rouxel, 2006 ▸; Dauphas *et al.*, 2017 ▸). Tin isotope systematics is an emerging stable isotope system (Wang *et al.*, 2017 ▸, 2018 ▸; Creech *et al.*, 2017 ▸; Brügmann *et al.*, 2017 ▸).

**Figure 11:**
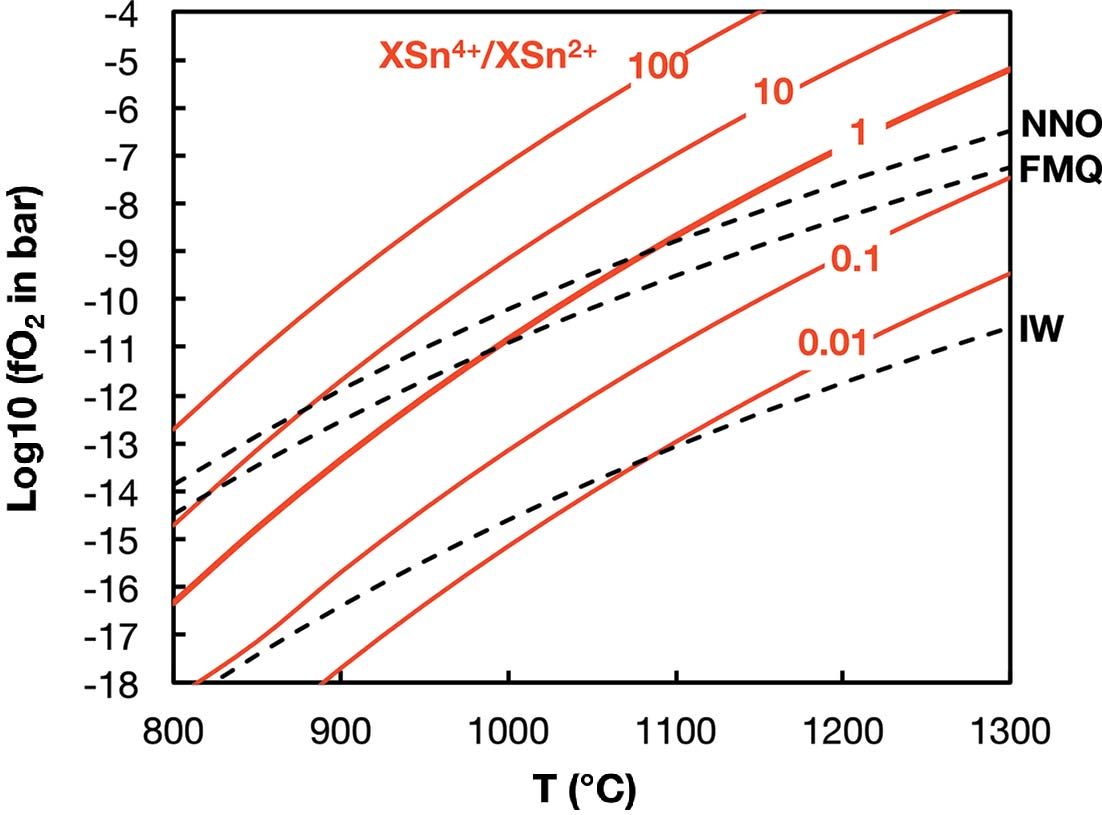
Proportions of Sn^4+^ and Sn^2+^ in Na_2_O.2SiO_2_ glass (Johnston, 1965 ▸) as a function of oxygen fugacity (fO_2_) and temperature [equation (66)]. Several oxygen buffers are shown; NNO = Ni–NiO, FMQ = fayalite–magnetite–quartz, IW = iron–wüstite. Most terrestrial igneous rocks have oxygen fugacities within ±2 Log-units of the FMQ buffer, where Sn^4+^ and Sn^2+^ could co-exist.

In aqueous systems, tin is predominantly Sn^4+^, except in acid and reducing environments, where Sn^2+^ can be present (Kabata-Pendias & Pendias, 2001 ▸) but there is clearly some potential to detect Sn isotopic fractionation. In magmatic systems, Sn^4+^ is a large highly charged ion that behaves as an incompatible element but can substitute for Fe^3+^ and Ti^4+^ in minerals. The SnO_2_–SnO buffer lies 2 to 4 log units below the FMQ (fayalite–magnetite–quartz) buffer, which taken at face value would indicate that Sn is dominated by Sn^4+^ in terrestrial magmas while Sn^2+^ could be present in significant amounts in reduced lavas (Lehmann, 2006 ▸). The activities of SnO_2_ and SnO in silicate melts could, however, affect the equilibrium between those two oxidation states. Indeed, the results of Johnston (1965 ▸) for Na_2_O·SiO_2_ glass put the Sn^4+^/Sn^2+^ equilibrium crossing the FMQ buffer at a temperature of 1000°C, meaning that Sn^2+^ could represent a significant portion of Sn in magmas. We used the data published in that paper to calculate the Sn^4+^/Sn^2+^ ratio in those glasses, taken as proxies of silicate melts,
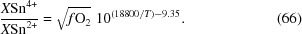
where 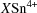 and 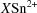 are the mol fractions of the two oxidation states of Sn, 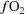 is the oxygen fugacity in bar, and *T* is the temperature in K. Most terrestrial rocks have oxygen fugacities within 2 log units of the FMQ oxygen buffer. As shown in Fig. 11 ▸, the rocks in that range are expected to display large variations in their Sn^4+^ and Sn^2+^ proportions. This, together with the fact that very large equilibrium isotopic fractionation is expected between these two oxidation states, means that the isotopic composition of Sn could be a useful tracer of redox processes in the Earth. Wang *et al.* (2018 ▸) speculated, for instance, that the heavy Sn isotopic composition of basalts relative to mantle peridotites could be due to the more incompatible behavior during mantle melting of isotopically heavy Sn^4+^ relative to light Sn^2+^. A similar idea had been proposed to explain the heavy Fe isotopic composition of basalts relative to peridotites (Dauphas *et al.*, 2009*a* ▸). More work is clearly needed to characterize the Sn^4+^/Sn^2+^ ratio of igneous rocks and evaluate how Sn isotopes are fractionated at equilibrium between these two oxidation states in silicate magmas.

We have also calculated the β-factor of solid Kr at 2 GPa, which gives
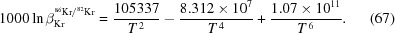
Because the 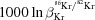 factor of mono-atomic gaseous Kr is 0, the formula above gives the equilibrium fractionation factor for Kr between solid and gas. Assuming that solid Kr at 2 GPa can be taken as a proxy for Kr trapped in ice at low *T*, we can calculate the isotopic composition of Kr in cometary ice at equilibrium with solar gas. The temperature of condensation of Kr in cometary ice is uncertain but a temperature of 50 K may be reasonable (Dauphas, 2003 ▸; Owen *et al.*, 1992 ▸; Mousis *et al.*, 2016 ▸). At such a temperature, we would predict that Kr in ice should be isotopically fractionated relative to the gas by +35‰ (3.5%) in the 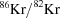 ratio. This value should be taken with a grain of salt but it shows that significant equilibrium isotopic fractionation may be present during trapping of Kr and possibly other noble gasses in cometary ice. As of today, there are no Kr isotope measurements of comets or experimental determinations with which to compare the calculated value.

### 4.4. A one-parameter expression for the temperature dependence of β-factors   

Dauphas *et al.* (2017 ▸) derived an approximate one-parameter formula to express the temperature-dependence of β-factors. This is useful in geochemistry to extrapolate equilibrium fractionation factors measured experimentally at one or a few temperatures to different temperatures (a 1/*T* ^2^ dependence is often assumed but the formulas given below yield more accurate extrapolations). If one assumes that the material has a Debye PDOS, the even moment of *g*(*E*) can be expressed as even powers of the Debye energy cutoff,
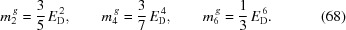
One can therefore approximate equation (58) by a one-parameter (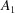) formula,
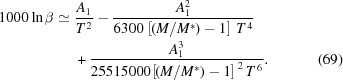
For the ^56^Fe/^54^Fe ratio, this would give
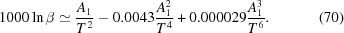
In practice, the PDOS of natural materials show large departures from a Debye behavior. Assuming a similar functional relationship as that derived for a Debye behavior in the more general case, one can write the β-factor as
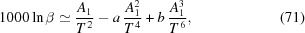
where *a* and *b* are fit parameters that can be calculated based on NRIXS measurements or *ab initio* studies that give the third-order polynomial expansion of the β-factor. For the ^56^Fe/^54^Fe ratio, Dauphas *et al.* (2017 ▸) obtained an approximate formula,
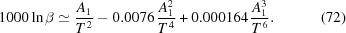
For the ^122^Sn/^116^Sn isotopic ratio, we use the three NRIXS data reported by Polyakov *et al.* (2005 ▸) to derive a one-parameter expression of the β-factor,
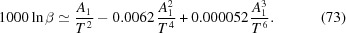
This formula will be refined as the database of NRIXS measurements continues to expand for Sn.

## 5. Conclusion   

The synchrotron radiation technique of nuclear resonant inelastic X-ray scattering is used across many fields, including material sciences, condensed matter physics, heme biochemistry, geophysics and isotopic geochemistry. Isotopic geochemistry requires accurate determination of the force constant of the chemical bonds, which depends on the third moment of the NRIXS spectrum and is prone to biases that had not been fully appreciated before. This prompted us to develop a new software called *SciPhon* to reduce NRIXS data for all applications. This software runs in *Mathematica* and is thus portable on Windows, MacOS, Linux and Raspbian platforms. It is also perennial as most *Mathematica* codes are portable in new versions of the software. A virtue of using the *Mathematica* platform is that a user-friendly GUI can be deployed on many operating systems. The program runs rapidly, with each step in the data processing not taking more than a few seconds. The total data reduction can be done in a minute or two. NRIXS data for all Mössbauer isotopes can be reduced with this software. The options offered to the user are those studied at the APS but this can easily be extended to include other Mössbauer isotopes. *SciPhon* presents several features that make it an asset when reducing NRIXS data:

(1) It has a GUI, which facilitates learning of the program and speeds up data processing for the most repetitive tasks.

(2) It provides flexibility in the definition of the baseline/background.

(3) It allows the user to define the energy range used in data reduction.

(4) It propagates uncertainties from counting statistics, as well as baseline subtraction and energy scaling.

(5) It outputs all the parameters that can be extracted from the NRIXS spectrum and partial PDOS. This includes a built-in function to calculate the Debye velocity from the PDOS as well as all data needed to calculate reduced partition function ratios used in isotope geochemistry.

(6) It uses an original approach to remove the elastic peak using an interpolation that respects the detailed balance.

Extensive testing was performed to ensure that the software performs as expected. *SciPhon* yields values that are most often consistent with the values derived by *PHOENIX*. The baseline subtraction procedure used in *SciPhon* has been proven to yield reproducible data. *SciPhon* is routinely used by our group and others and data generated by it have already been published in top journals (Dauphas *et al.*, 2014 ▸; Blanchard *et al.*, 2015 ▸; Roskosz *et al.*, 2015 ▸; Shahar *et al.*, 2016 ▸; Liu *et al.*, 2017 ▸).

## Supplementary Material

CSV file containing the PDOS values for all the compounds examined in the paper. DOI: 10.1107/S1600577518009487/fv5085sup1.txt
